# Growth performance, carcass characteristics and meat sensory evaluation of broiler chickens fed diets with fermented cassava leaves

**DOI:** 10.5713/ab.23.0362

**Published:** 2024-01-20

**Authors:** Arti Bhavna, Titus J. Zindove, Paul A. Iji, Archibold G. Bakare

**Affiliations:** 1 Department of Animal Science, School of Animal and Veterinary Sciences, Fiji National University, Nasinu, Fiji Islands; 2 Faculty of Agriculture and Life Sciences, Lincoln University, Lincoln 7647, New Zealand; 3 School of Environmental and Rural Sciences, University of New England, Armidale 2350, Australia

**Keywords:** Broiler Chickens, Cooking Methods, Fermented Cassava Leaves, Growth Performance, Meat Quality Evaluation, Silage Additives

## Abstract

**Objective:**

The objective of the study was to determine the effects of feeding fermented cassava leaf meal (FCLM) on growth performance, carcass characteristics and meat sensory evaluation of broiler chickens.

**Methods:**

A total of 160 Cobb-500 chickens were used during the phases of growing (21 days of age; initial weight 0.39±0.025 kg/bird) and finishing (35 days of age; initial weight 1.023±0.164 kg/bird). The whole experiment lasted for four weeks. The FCLM was included in starter and finishing diets at 0, 50, 100, and 150 g/kg inclusion levels. Total feed intake (TFI), weight gain (WG), feed conversion ratio, and nutrient digestibility were recorded. Sensory evaluation of breast meat was used to determine the eating quality of the meat prepared using roasting and boiling methods.

**Results:**

The TFI and WG decreased (p<0.05) with increasing inclusion levels of FCLM in the diets of growing chickens. Crude protein digestibility for chickens fed 0 and 50 g/kg FCLM was higher (p<0.05) than for chickens subjected to a diet with 150 g/kg FCLM. During the finishing phase, TFI increased linearly (p<0.05) with increasing inclusion level of FCLM in chicken diets, while WG decreased (p<0.05) with inclusion level of FCLM. Treatment diets had no effect (p>0.05) on the eating qualities of breast meat. However, juiciness was significant (p<0.05) for the cooking method and treatment interaction. At 50 g/kg inclusion level, boiled meat had a higher (p<0.05) juiciness score than roasted meat. Tenderness, on the other hand, was significant (p<0.05) for the interaction of gender and treatment. Females considered the boiled meat to be more tender than the males at 150 g/kg inclusion level. Using principal component analysis, a positive correlation was observed between teeth adhesion and fibrousness, flavour and juiciness, and springiness and tenderness.

**Conclusion:**

From the study, it can be concluded that FCLM can be used as an ingredient in the diets of broiler chickens. Inclusion level of 50 g/kg can be used in chicken diets during the growing phase, whereas in the finishing phase, inclusion level of 150 g/kg FCLM can be used. The FCLM did not affect the eating quality of breast meat.

## INTRODUCTION

A study by Bakare et al [1] has revealed that up to 100 g/kg of cassava leaf meal can be included in diets without causing any deleterious effects on the chickens. The quantities of leaf meal in chicken diets are limited due to fibre levels and concentration of anti-nutritional factors. There is, however, a possibility that using fermented cassava leaf meal (FCLM) will likely improve the growth performance of chickens. Fermentation reduces hydrogen cyanide to innocuous levels, and it is also believed that fibres are reduced, and a sweet smell is imparted on the feed material [2,3]. This will likely increase intake and, consequently, the overall performance of the chickens. However, cassava leaves are not fermentable material because they have low water-soluble carbohydrates [4]. Therefore, additives like *Lactobacillus buchneri* and molasses will provide the catalyse and/or substrate to improve fermentation. To our knowledge, information on the different inclusion levels of FCLM in diets on the growth performance of chickens is scarce. Hence, it warrants further investigation.

Diets have also been noted to affect the carcass characteristics of chickens [5]. Some examples of these characteristics are carcass weight and carcass cuts, such as breast meat, drumstick, wings and thighs [6]. In addition, some visceral organs, such as the liver, gizzard, heart, intestines and spleen, are also important. The visceral organs are a delicacy in most developing countries, including Fiji. We postulate that FCLM inclusion levels up to 150 g/kg can be added to poultry diets without affecting overall performance. The use of FCLM will help reduce feed costs, a problem faced by most farmers in the Pacific region.

The physical properties of the meat are directly influenced by the diet that broiler chickens consume [7]. These properties include, but are not limited to, sensory properties like flavour, juiciness and tenderness. There is a high probability that feeding different inclusion levels of FCLM will also likely affect the sensory properties and/or eating quality of meat. Roasting and boiling are commonly used chicken preparation methods in the South Pacific islands [8]. Population in Fiji differ in income levels; thus, the choice of the method of meat preparation depends on the availability and cost of equipment. Consumers who cannot afford to buy roasting equipment can opt to boil the meat, whereas those who can afford it can use ovens for roasting. These methods will affect the organoleptic characteristics of the meat [9]. Sensory evaluation is important to test if the meat from chickens fed FCLM diets will be accepted by different types of consumers. The objective of the study was, therefore, to determine the effects of feeding FCLM on growth performance, carcass characteristics and meat sensory evaluation of broiler chickens.

## MATERIALS AND METHODS

### Study site

All experiments and procedures in this study were approved by the Animal Ethics (FNU-AREC-22-001) and the Human Ethics (FNU-HREC-22-42) Committees of the Fiji National University. The study was conducted on the Fiji National University farm, about 13 kilometres north of Suva. The farm is located between latitudes 15° to 20° S and 175° to 182° E. The pens were built with concrete flooring and mesh wire walls that were adequately ventilated. Data loggers were used for recording average ambient temperature and relative humidity. The experimental period’s average temperature and relative humidity were 27.1°C±1.23°C and 82.3%±3.32%, respectively.

### Harvesting and processing of cassava leaves and diet formulation

Mature cassava leaves were hand-harvested from the Fiji National University crop farm. These leaves were harvested with minimum plant disturbance, enabling shoots to regrow. The stalk from the leaves was removed, and after that, the leaves were chopped to 1 to 3 cm using a shredder. The chopped leaves were fermented with *Lactobacillus buchneri* (Bio-way Technology Co., Ltd., Shanghai, China) and molasses to increase the fermentation rate. *Lactobacillus buchneri* was inoculated in the cassava leaves at a concentration of 3.1×10^8^ cfu/mL. Approximately 100 mL of inoculant was added to 25 kg fresh forage. Molasses was added at a level of 5 g/100 g. Silo drums were used to store the chopped leaves to avoid environmental interference. The fermentation process lasted one month. The fermented leaves were then sun-dried until they became crisp. After that, the dried leaves were ground with a milling machine to pass through a 2-mm sieve, and samples were collected for chemical analyses. Table 1 shows the nutrient composition of FCLM, which was utilised to formulate feed.

Four diets were formulated with FCLM incorporated at four different inclusion levels of 0, 50, 100, and 150 g/kg dry matter (DM) in chicken diets. In addition, other ingredients such as soybean meal, copra, wheat, wheat middlings and corn were used to formulate the different diets to meet birds’ nutritional needs. The diets were presented to the chickens in mash form. Table 2 shows the ingredients and nutrient composition of the diets used in the experiment. The crude fibre (CF) content of the treatment diets increased with inclusion level FCLM. It was assumed that fibre acts as a diluent and reduces nutrient density in diets [10], and that the chickens would eat more to compensate for the reduced nutrient density. Crude protein (CP), CF, crude fat and gross energy were analysed using procedures described in AOAC [11].

### Birds, feeding and experimental design

Two hundred Cobb 160-day-old chicks were used during the phases of growing (21 days of age; initial weight 0.39± 0.025 kg/bird) and finishing (35 days of age; initial weight 1.023±0.164 kg/bird). The whole experiment was conducted for four weeks. A total of 32 pens housing five chickens each were used for the experiment. Each pen was used as the experimental unit. The feeding trial had four treatments and eight replicates altogether. Each chicken group was randomly assigned to the treatment diets (0, 50, 100, 150 g/kg FCLM). A total of 40 chicks per treatment diet were used in the study. Feed and water were provided ad-libitum. The chickens were exposed to 12 hours of natural light per day during the growing and finishing phase.

### Measurement of growth performance parameters

Growth performance parameters measured in the experiment were total feed intake (TFI), weight gain (WG), and feed conversion ratio (FCR). Feed intake was recorded daily every morning. Feed spillage was also taken into consideration. Feed left in the feed trough was weighed daily. This information was used to calculate the average daily feed intake of birds.

### Nutrient digestibility

On day 30, at least three birds per replicate were euthanised by cervical dislocation and excised. To estimate apparent ileal digestibility (AID), ileal digesta samples from each pen were pooled. Ileal digesta was collected and stored in a freezer (−20°C) prior to analyses of nutrient composition. The experiment used Celite, a major acid-insoluble ash (AIA) component, as a digestibility marker. The concentration of AIA in the diet and digesta was measured and determined as described by Choct et al [12]. The digestibility coefficients were converted to percentages. The digestibility coefficient of the nutrients was calculated using the formula:


$$\begin{array}{l} \text{Apparent ileal digestibility coefficient }(\text{CP})\\ =1-\frac{\text{Ileal nutrient }(\text{g}/\text{kg})/\text{Ileal AIA }(\text{g}/\text{kg})}{\text{Diet nutrient }(\text{g}/\text{kg})/\text{Diet AIA }(\text{g}/\text{kg})} \end{array}$$

Dry matter digestibility was estimated using the following formula:


$$\begin{array}{l} \text{Dry matter digestibility }(\%)\\ =\frac{DMdiet-\left(\frac{Diet\;AIA\;(\frac{g}{kg})}{Ileal\;AIA\;(\frac{g}{kg})}\times DM\;Ileal\right)}{DM\;diet}\times 100 \end{array}$$

### Measurement of carcass characteristics

After slaughtering and bleeding the chickens, the carcasses were scalded in water for 30 seconds at 65°C before de-feathering. Later, the dressed chicks were eviscerated. Dressed weight percent, thigh, drumstick, shank, breast muscle, wing, and internal organ (gizzard, liver, heart, spleen, and small intestine) were measured on ten eviscerated carcasses per replicate. Every sample was measured thrice, and the average value was used to determine the outcome [13]. The relative weight of the organs was then determined using the body weight at slaughter [14].

### Preparation and cooking of meat

#### Collection of meat samples

One hundred and sixty broiler chickens were slaughtered to determine the eating quality of meat. The breast muscles (Pectoralis major) were removed from the chicken carcass, packed in vacuum-sealed bags and stored in a freezer at −18°C. All breast meat samples were thawed overnight at 4°C before cooking for evaluation. The portions of breast meat from chickens fed diets with different inclusion levels of FCLM were weighed in separate bowls. Boiling and roasting methods were used to determine the eating quality of the meat.

#### Cooking of meat samples

A random sample of breast muscles from each treatment (0, 50, 100, and 150 g/kg) was chosen and cooked for sensory evaluation of meat. Meat from each treatment was placed in separate bowls, and salt was added to taste at a rate of 2 g/100 g of meat. The salt was thoroughly mixed with the meat before being roasted in the oven. The meat was roasted at 200° in different aluminium pans until an internal temperature of 80° was achieved [8]. After that, the meat was removed from the oven, wrapped in foil, and kept warm until serving. The second method of preparation was boiling. The boiling method was used according to the procedure described by Jeon et al [15]. The breast muscle samples were heated in a pot with water (1.5 times the breast weight) using a gas burner, and salt (2 g/100 g meat) was added to taste. The samples were heated for 1 hour with maximum heat for 30 minutes and then minimum heat for another 30 minutes. The meat was prepared separately by treatments. The cooked meat samples were kept in a food warmer until served. Before serving, the roasted and boiled meat samples of breast muscles were cut into approximately equal sizes (1.5 cm×1.5 cm×1.5 cm), placed on plates and served with filtered water.

### Consumer sensory evaluation

A total of 20 trained male and female panellists of university staff and students evaluated the cooked meat samples. All panellists were advised about the experiment without disclosing the identity of the samples. Sensory panel booths for meat evaluation were used. Each panellist received two to three portions of breast meat from chickens fed different treatment diets. There were two sensory evaluation sections. In the first session, the panellists tasted meat samples prepared by roasting, and in the second session, the meat was prepared by boiling. A resting time of half an hour was given to the panellists between sessions to prevent biased results for the different cooking methods. To avoid prejudice in the rating procedure and to clear the taste receptors, filtered water was offered. The attributes such as tenderness, juiciness, springiness, teeth adhesion, flavour and fibrousness were investigated as described by Bakare et al [8].

### Statistical analysis

The Proc general linear model procedure of SAS [16] was used to analyse data for the growth performance of chickens in the growing and finishing phase, carcass characteristics and sensory evaluation score data. The following models were used for the analyses:


$$\text{Y}=\mu +\alpha_{\text{i}}+{\dot{\text{e}}}_{\text{ij}}$$

Where; Y, growth performance (feed intake, WG, FCR, and nutrient digestibility parameters) and carcass characteristics (weight of carcass, thigh, drumstick, wings, shanks, breast muscles and internal organs); μ, mean population; α_i_, effect of inclusion level (i = 0, 50, 100, 150 g/kg); ė_ij_, error term.

The quadratic response surface model [16] was used to determine the relationships between inclusion level and response variables (average daily feed intake, WG, FCR, and nutrient digestibility).

Model used for analysing the sensory evaluation scores data was:


$$\text{Y}=\mu +\alpha_{\text{i}}+\beta_{\text{j}}+{\text{c}}_{\text{k}}+{\left(\alpha \beta \text{c}\right)}_{\text{ijk}}+{\dot{\text{e}}}_{\text{ijkl}}$$

Where; Y_ijkl_, scores for sensory attributes (tenderness, juiciness, springiness, teeth adhesion, flavour and fibrousness; μ, mean population; α_i_, cooking method (i = roasting and boiling); β_j_, gender (j = male and female); c_k_, effect of inclusion level (k = 0, 50, 100, 150 g/kg); (αβc)_ijk_, effect of two-way interactions; ė_ijkl_, error term.

The pdiff option of SAS [16] was used for mean separation at p<0.05. The mean scores of sensory characteristics of the meat from chickens fed FCLM diets were subjected to principal component analysis (PCA) to determine the relationships among the sensory characteristics and the projection of estimates for the panellists of a different gender for samples from the four treatment groups.

## RESULTS

The effects of feeding chicken diets with different inclusion levels of FCLM on the growth performance of broiler chickens are shown in Table 3. The TFI during the growing phase was significant (p<0.05) for chickens fed diets with FCLM. The TFI was higher for chickens fed 0, 50, and 100 g/kg of FCLM than for chickens fed 150 g/kg FCLM. Weight gain decreased linearly (p<0.05) with inclusion level of FCLM in chicken diets. The FCR was higher for chickens fed 0, 50, and 100 g/kg of FCLM and lowest in chickens fed 150 g/kg FCLM. During the finishing phase, TFI was higher (p<0.05) in chickens fed 150 g/kg FCLM than in chickens fed a diet without FCLM. The TFI increased linearly (p<0.05) with inclusion level of FCLM in chicken diets. There was no significant relationship between WG and inclusion level of FCLM in chicken diets. Feed conversion ratio values were lower (p<0.05) for chickens fed diets with 150 g/kg FCLM than other treatment diets. Overall, TFI was not significant for chickens fed FCLM. Weight gain was higher (p<0.05) for chickens fed diets with 0 and 50 g/kg FCLM than those fed on diets with 100 and 150 g/kg FCLM. Weight gain of the chickens decreased linearly (p<0.05) with increasing inclusion levels of FCLM. Feed conversion ratio values were higher (p<0.05) for chickens fed 100 and 150 g/kg of FCLM than those fed diets with 0 and 50 g/kg FCLM.

Table 4 shows the AID of DM and crude protein. The DM digestibility coefficient was lower (p<0.05) for chickens fed 150 g/kg FCLM diets than those subjected to other treatment diets. On the other hand, crude protein digestibility for chickens fed 0 and 50 g/kg FCLM was higher (p<0.05) than for chickens subjected to a diet with 150 g/kg FCLM.

**Figure 1:**
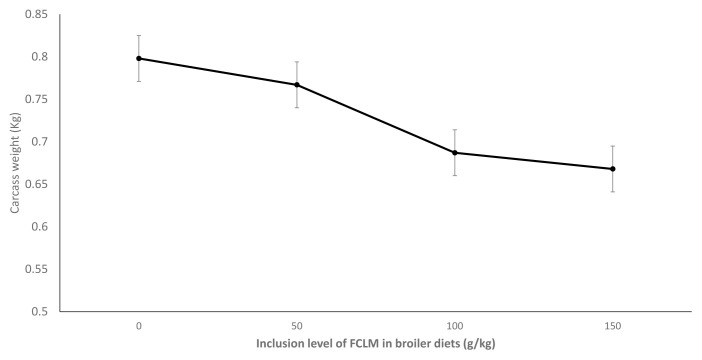
Effect of feeding graded levels of fermented cassava leaves (FCLM) in broiler diets on carcass weights of broiler chickens.

The effects of feeding graded levels of fermented cassava leaves on carcass weights of broiler chickens are shown in Figure 1. Carcass weights of chickens decreased (p<0.05) with inclusion level of FCLM. Higher carcass weights were observed in chickens fed 0 and 50 g/kg FCLM than in chickens fed 100 and 150 g/kg FCLM.

Table 5 shows the relative weights of meat cuts and offals of broilers fed diets with different inclusion levels of FCLM. The relative weights of thighs, drumsticks and breasts were not significant for chickens fed diets with different inclusion levels of FCLM. There was a difference (p<0.05) in the relative weights of wings from chickens fed different inclusion levels of FCLM. However, there was no clear-cut trend on how different inclusion levels of FCLM affected the wings. The same was noted for the relative weights of hearts.

The summary statistics of sensory evaluation parameters are shown in Table 6. The use of cooking methods significantly affected meat tenderness and fibrousness (p<0.05). At the same time, springiness and juiciness also had a significant response (p<0.05) when gender was used as an independent variable. The interaction of the cooking method and treatment had an effect (p<0.05) on the juiciness of meat from chickens fed FCLM. Interaction of gender and treatment, on the other hand, had an effect (p<0.05) on tenderness of meat from chickens fed FCLM.

**Figure 2:**
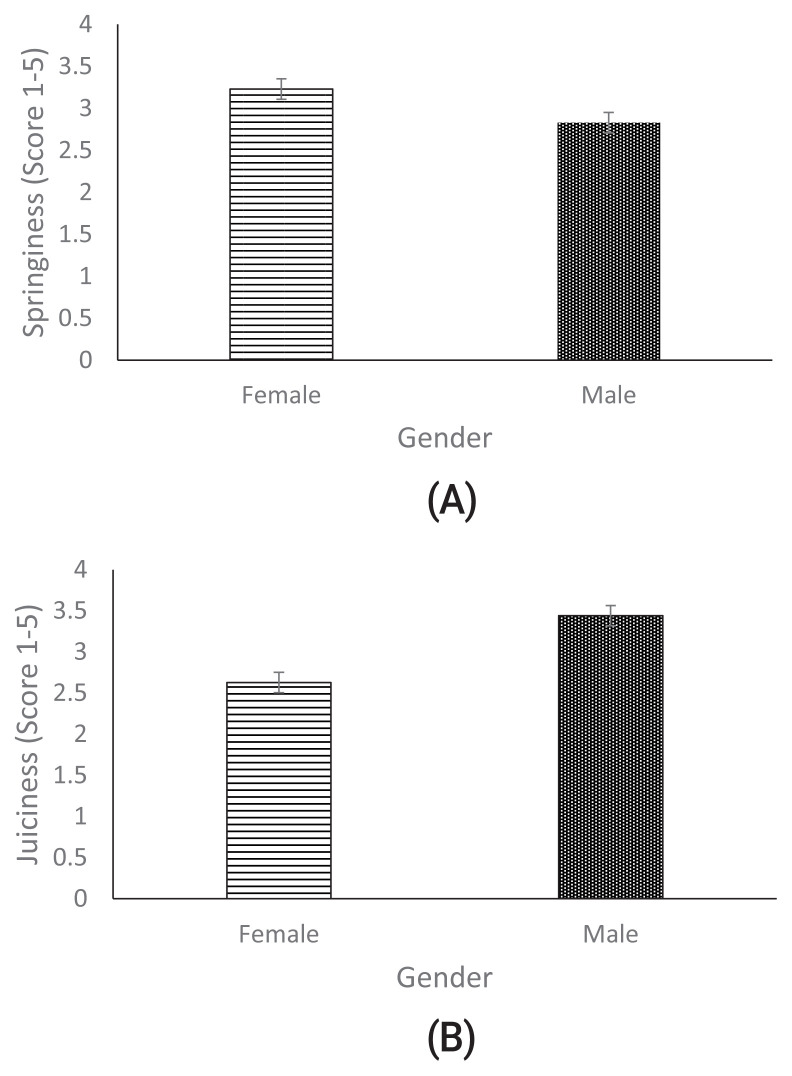
Effect of gender on springiness (A) and juiciness (B) of meat from chickens fed fermented cassava leaf meal.

Table 7 shows the effects of cooking methods on different sensory attributes of breast meat from chickens fed FCLM. Springiness, juiciness, flavour and teeth adhesion were not significant for either of the cooking methods. Roasted meat was regarded as more tender than boiled meat (p<0.05). For fibrousness, roasted meat was again regarded as more fibrous than boiled meat (p<0.05). The effects of gender on the springiness and juiciness of meat from chickens fed graded levels of FCLM are shown in Figure 2. Males recorded a low level of springiness of meat compared to females, who recorded a high degree of springiness (p<0.05). Sensory scores for meat juiciness were higher (p<0.05) for males than females.

Table 8 shows the interaction of treatment and cooking methods on the juiciness of meat from chickens fed FCLM. Inclusion levels of 0, 100 and 150 g/kg FCLM were not significant for the different cooking methods. At 50 g/kg inclusion level, there was a difference (p<0.05) in the juiciness of meat using boiling and cooking methods. Boiled meat had a higher (p<0.05) juiciness score than roasted meat. Table 9 shows the interaction of treatment and gender on meat tenderness from chickens fed FCLM. The inclusion level of 100 g/kg FCLM was not significant for gender. For 0, 50, and 150 g/kg inclusion levels, there was a difference (p<0.05) in the eating quality of meat for both males and females. For the inclusion level of 0 and 50 g/kg of FCLM, males scored the cooked meat more tender than females. At 150 g/kg inclusion level, females considered the cooked meat samples more tender than the males.

**Figure 3:**
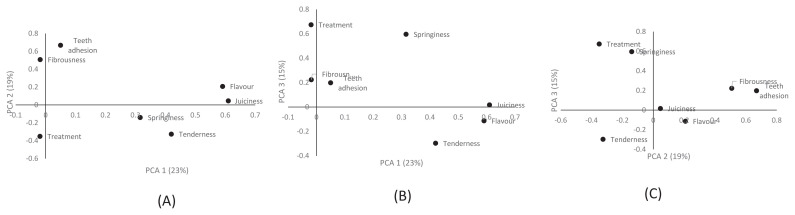
Principal component analysis (PCA) for relationships among the sensory properties of breast meat. (A) PCA 1 vs PCA 2, (B) PCA 1 vs PCA 3, and (C) PCA 2 vs PCA 3.

**Figure 4:**
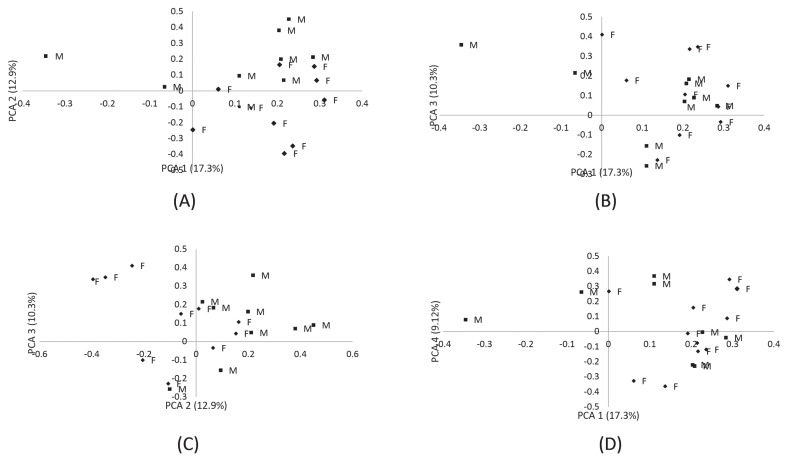
Prediction of twenty panelists for samples from four treatment groups (0, 50, 100, and 150 g/kg inclusion levels) based on principal component analysis (PCA) derived from gender and cooking methods. F, females; M, males.

Figure 3 shows the relationships between treatment and sensory evaluation parameters of breast muscles from chickens fed FCLM diets. The relationships can be described by PCA 1, PCA 2, and PCA 3, which accounted for 57% of the variation. A positive correlation was observed between teeth adhesion and fibrousness, flavour and juiciness, and springiness and tenderness. Treatment was not related to the sensory attributes. Figure 4 interprets data from panellists (n = 20) given breast muscles from chickens fed FCLM-based diets. The results show no difference in patterns for scoring sensory parameters between male and female panellists.

## DISCUSSION

A preliminary study was conducted to determine how ensiling can affect the physicochemical properties of cassava leaves [17]. In the study, it was observed that silage with better physical characteristics was ensiled by inoculating *Lactobacillus buchneri* at concentrations of 3.1×10^10^ cfu/mL and molasses at a concentration of 5 to 7 g/100 g. The concentrations of bacteria and molasses used in the study resulted in silage with better physical characteristics, and no moulds or signs of spoilage were observed.

Voluntary feed intake is among the most crucial elements affecting an animal’s performance. The observation that TFI and WG were low for chickens fed diets with higher inclusion levels of FCLM (150 g/kg) during the growing phase might be attributed to the high fibre content and anti-nutritional factors in diets with higher inclusion levels of FCLM. In the study, increasing the inclusion level of FCLM increased the fibre content of the diet. Hence, high inclusion levels of FCLM might have contributed to the bulkiness of feed, which resulted in insufficient intake of nutrients, particularly protein and energy required to sustain rapid growth. Melesse et al [18] reported that higher inclusion levels of cassava leaf meal reduced feed intake in chickens. Eruvbetine et al [19] reported similar findings; feed intake was reduced at higher cassava leaf meal inclusion levels in chicken diets. Due to this, the nutrients may not be readily available for use by the animal. Thus, a decline in body weight was observed in chickens fed diets with high inclusion levels of FCLM. If immature/young leaves with low fibre content had been used, which was not the case in this study, better results on performance would have been achieved. The low FCR during the growing phase was expected, considering chickens fed diets with FCLM showed low TFI and WG. Also, the observed crude protein digestibility for chickens fed diets with inclusion levels of 0 and 50 g/kg FCLM depicts that the nutrients were well assimilated and used up by the chickens. Hence, more WG was observed in chickens fed diets with 0 and 50 g/kg FCLM.

The observed difference in TFI and WG patterns between the growing and finishing phases might be attributed to the five-day adaptation period before the experimental trial. The decrease in TFI during the growing phase of this trial was probably due to the less time given to the chickens to adjust to the FLCM diets. We assume that the growing bird’s immature gut did not support the transit of digesta with the same efficiency as that of older birds; hence, intake decreased with the inclusion level of FCLM in growing chickens. An increase in intake with inclusion level of FCLM during the finishing phase was expected. According to Bakare et al [1], the fibre content in the feed acts as a diluent with negative implications on feed intake, nutrient density and nutrient digestibility. To compensate for the reduced density of nutrients, there was an increase in TFI during the finisher phase of the trial to meet the nutritional requirements. The observation that overall TFI did not vary among the treatments conforms to studies that reported chickens to have a low taste acuity [20]. Hence, volatile fatty acids and some anti-nutritional factors do not adversely affect chickens. Volatile fatty acids and anti-nutritional factors in the study were, however, not measured, and thus, this warrants further investigation.

The decrease in the proportion of thighs, drumsticks, wings and breasts with the addition of leaf meal in the study could be related to the decrease in carcass weight with increasing levels of FCLM fed to chickens. According to Musa et al [21] and Park et al [22], the weight of the chicken parts is positively correlated to the weight of the carcass. This is also supported by Ncube et al [23], who fed *Acacia angustissima* leaf meal in broiler diets and observed a similar trend.

In our study, we expected the relative weights of gizzards to increase with inclusion level of FCLM in the chicken diets. This is mainly because of the nature of the feed offered to the chickens. The fibre content of the feed increased with inclusion level of FCLM. The volume of feed, the length of time spent grinding the feed, and the frequency of gizzard contractions, which are required to process the large particles for further digestion in the distal sections of the intestine, are factors expected to increase the weights of gizzards [24]. The liver responds to diets by producing prodigious fluids for digestion [25]. Hence, we were expecting changes in the relative weights of the livers. This was not the case in our study, as no differences were observed among the treatments.

Animal diet directly influences muscle characteristics [26]. In our study, the chickens were fed different dietary treatments; hence, we expected differences in the muscle characteristics of meat samples. In addition, muscle characteristics affect meat’s water-holding capacity and juiciness [27]. Thus, the observed differences in juiciness for meat from chickens subjected to different treatment diets in our study were expected. Muscle characteristics and water-holding capacity of meat in our study were, however, not measured. These results align with Navid et al [28] study, which reported that feeding spent layer hens 2% papaya leaf meal affects the meat’s tenderness and juiciness. Other studies showing significant effects of leaf meals on the juiciness of meat were by Ncube et al [23], Marangoni et al [29], Abdelatty et al [30] and Sakr et al [31].

The observation that tenderness was given a higher score for roasting than the boiling method was not expected. Abdalla et al [32] found that moist cooking had a greater effect on the tenderness of chicken meat than the oven cooking method. Wołoszyn et al [33] used different cooking methods for preparing goose meat. They found that meat samples boiled in a water bath were tender compared to other cooking methods like oven convection roasting, grilling and pan-frying. A similar finding was reported by Ježek et al [34], who reported boiled meat samples to attain a greater level of tenderness. In our study, boiled meat was tough, which might be attributed to the fact that we boiled the meat at a high temperature for 30 minutes. According to Dransfield [35], boiling meat at higher temperatures results in faster rigour development, causing it to be tough. Hence, this conforms with our findings. We assume the cooking method might have a masking effect on consumer perceptions of meat, resulting in treatment not affecting most sensory evaluation parameters. There is a need for conducting analytical studies to determine how feeding of FCLM affects the physical and chemical properties of meat.

The observation that the fibrousness of roasted breast muscles was given a higher score than boiled meat samples might be because breast muscles consist of a thin muscle loosely attached to the underside of the breast that increases the fibre content when the roasting method is used [36]. Hence, we assume that roasting of breast muscles leads to water loss, thus causing the cooked meat to harden, showing its fibrous content of meat. This might have been the case in our study. Hence, further studies must confirm this result.

The observation from our study revealed that males gave a higher score for juiciness than females. This might be due to the fact that gender variations in sensory acuity may alter food choices since women are more sensitive to sweet and salty tastes, which could explain why males gave juiciness a higher score. A similar finding was reported by Bartlett and Beckford [37], who used sweet potato root meal (SPRM) and found that males scored meat from 30% SPRM diets to be juicier when compared to meat from birds fed 20% SPRM diets. Overall, from our study, there were not many variations in scores given by males and females.

## CONCLUSION

From the study, it can be concluded that FCLM can be fed to chickens. Inclusion levels of 50 g/kg can be used in chicken diets during the growing phase, whereas in the finishing phase, inclusion levels of 150 g/kg FCLM can be used without compromising the performance of chickens. Our findings also indicate that adding FCLM to chicken diets does not affect the eating qualities of breast meat prepared using different cooking methods. Future research on the amino acid profiles of FCLM is required to assess the protein quality of FCLM.

## Figures and Tables

**Table 1:** Chemical composition of fermented cassava leaf meal

Parameter	Composition
Crude protein (g/100 g)	17.5
Crude fibre (g/100 g)	10.8
Ether extract (g/100 g)	6.7
Gross energy (kJ/100 g)	1,472

**Table 2:** Ingredients and chemical composition of broiler grower and finisher diets

Items	Grower				Finisher			
	
	Inclusion level (g/kg)				Inclusion level (g/kg)			
	
	0	50	100	150	0	50	100	150
Ingredient (%)								
Corn	16	16	16	16	16	16	16	16
Soybean	27	25.9	24.2	23	17.5	16	15	15
Wheat middlings	4	5	6.4	6	3	7	8	8
Salt	1	1	1	1	1	1	1	1
Wheat grains	40	37.1	32	28	43	35	29	24
Copra	12	10	10.4	11	19.5	20	21	21
FCLM	0	5	10	15	0	5	10	15
Vitamin mix^1)^	+	+	+	+	+	+	+	+
Mineral mix^2)^	+	+	+	+	+	+	+	+
DCP^3)^	+	+	+	+	+	+	+	+
Total	100	100	100	100	100	100	100	100
Chemical composition								
CP	21.96	21.88	21.92	21.98	19.05	19.13	19.17	19.77
ME	1,417.3	1,418.18	1,419.88	1,418.89	1,460.86	1,466.66	1,465.82	1,469.74
Fiber	3.72	4.31	5.14	5.84	3.93	5.35	6.24	7.01

FCLM, fermented cassava leaf meal; DCP, dicalcium phosphate; CP, crude protein; ME, metabolizable energy.

1) Minerals premix supplied per 0.7 kg/tonne of diet: mineral oil, 2.5 mg; Cu (sulphate), 16 mg; Fe (sulphate), 40 mg; I (iodide), 1.25 mg; Se (selenate), 0.3 mg; Mn (sulphate and oxide), 120 mg; Zn (sulphate and oxide), 100 mg; cereal-based carrier, 128 mg; mineral oil, 3.75 mg.

2) Vitamin premix supplied per 0.5 kg/tonne of diet: retinol, 12,000 IU; cholecalciferol, 5,000 IU; tocopheryl acetate, 75 mg; menadione, 3 mg; thiamine, 3 mg; riboflavin, 8 mg; niacin, 55 mg; pantothenate, 13 mg; pyridoxine, 5 mg; folate, 2 mg; cyanocobalamin, 16 μg; biotin, 200 μg; cereal-based carrier, 149 mg.

3) Dicalcium phosphate (DCP) application rate 200 g/70 kg feed: composition 100 g contain – 24 g (Calcium); 18 g (Phosphorus); 2 g (Aluminium); 0.1 g (Fluorine).

**Table 3:** Effect of feeding graded levels of fermented cassava leaves on growth performance of broiler chickens

Feed type	Inclusion level (g/kg)				L^1)^	Q^1)^

	0	50	100	150		
Grower						
TFI	0.92±0.022^a^	0.86±0.018^a^	0.87±0.026^a^	0.78±0.024^b^	^*^	NS
WG	0.55±0.022^a^	0.45±0.023^b^	0.42±0.020^c^	0.28±0.040^d^	^*^	NS
FCR	1.68±0.039^a^	1.94±0.057^a^	2.10±0.066^a^	3.47±0.31^b^	NS	^*^
Finisher						
TFI	0.94±0.032^a^	0.98±0.034^ab^	0.96±0.033^ab^	1.05±0.0422^b^	^*^	NS
WG	0.17±0.026^a^	0.27±0.046^ab^	0.19±0.035^a^	0.33±0.054^b^	NS	NS
FCR	6.11±0.33^a^	6.71±1.267^a^	5.97±0.332^a^	3.68±0.222^b^	NS	^*^
Overall						
TFI	1.86±0.046^a^	1.85±0.045^a^	1.84±0.047^a^	1.83±0.056^a^	NS	NS
WG	0.73±0.038^a^	0.73±0.055^a^	0.61±0.042^b^	0.61±0.030^b^	^*^	NS
FCR	2.59±0.064^a^	2.62±0.080^a^	3.09±0.098^b^	3.04±0.087^b^	^*^	NS

TFI, total feed intake; WG, weight gain; FCR, feed conversion ratio.

1) L, linear; Q, quadratic.

* Significant; NS, not significant.

a–d Means sharing the same superscript within the same row are not different (p>0.05).

**Table 4:** Apparent ileal digestibility of dry matter and crude protein for chickens fed graded levels of FCLM

FCLM inclusion level (g/kg)	Dry matter digestibility (%)	Crude protein digestibility (%)
0	58.45±1.834^a^	75.81±1.673^a^
50	60.81±1.863^a^	71.24±0.960^ab^
100	59.05±0.672^a^	73.80±0.973^b^
150	45.83±0.372^b^	62.31±0.381^c^

FCLM, fermented cassava leaf meal.

a–c Means sharing the same superscript within the same column are not different (p>0.05).

**Table 5:** Effect of feeding graded levels of fermented cassava leaves on relative organ weights of broiler chickens

Items	Inclusion level (g/kg)				L^1)^	Q^1)^

	0	50	100	150		
Meat parts						
Thighs	0.153±0.005^a^	0.156±0.007^a^	0.171±0.008^a^	0.160±0.005^a^	NS	NS
Drumstick	0.153±0.005^a^	0.161±0.007^a^	0.170±0.009^a^	0.159±0.006^a^	NS	NS
Wings	0.123±0.004^b^	0.129±0.005^ab^	0.138±0.005^a^	0.133±0.005^ab^	NS	NS
Breasts	0.216±0.009^a^	0.224±0.009^a^	0.235±0.011^a^	0.228±0.009^a^	NS	NS
Offals						
Gizzard	0.041±0.002^a^	0.043±0.002^a^	0.045±0.003^a^	0.045±0.001^a^	NS	NS
Liver	0.038±0.001^a^	0.041±0.002^a^	0.042±0.001^a^	0.038±0.001^a^	NS	NS
Heart	0.009±0.0003^b^	0.009±0.0005^ab^	0.011±0.0007^a^	0.009±0.0003^b^	NS	^*^

1) L, linear; Q, quadratic.

* Significant (p<0.05); NS, not significant.

a,b Means sharing the same superscript within the same row are not different (p>0.05).

**Table 6:** Summary statistics for sensory evaluation parameters

Dependent variable	Independent variables				

	Method	Gender	Treatment	Method×treatment	Gender×treatment
Tenderness	^*^	NS	NS	NS	^*^
Springiness	NS	^*^	NS	NS	NS
Juiciness	NS	^*^	NS	^*^	NS
Flavour	NS	NS	NS	NS	NS
Fibrousness	^*^	NS	NS	NS	NS
Teeth adhesion	NS	NS	NS	NS	NS

* Significant (p<0.05); NS, not significant.

**Table 7:** Effects of cooking methods on different scores for sensory attributes of breast meat from chickens fed fermented cassava leaf meal

Attributes	Boiling	Roasting	Standard error
Tenderness	3.04^a^	3.58^b^	0.116
Springiness	3.07^a^	2.99^a^	0.122
Juiciness	3.019^a^	3.056^a^	0.124
Flavour	3.13^a^	3.11^a^	0.129
Fibrousness	3.03^a^	3.51^b^	0.110
Teeth adhesion	2.83^a^	2.58^a^	0.123

a,b Means sharing the same superscript within the same row are not different (p>0.05).

**Table 8:** Effects of interaction of treatment and cooking methods on the juiciness of meat

Treatment	Boiling	Roasting
0	2.70^a^	3.20^a^
50	3.43^b^	2.58^b^
100	3.18^a^	3.58^a^
150	2.78^a^	2.88^ab^
Standard error (SE)	0.249	0.249

a,b Means sharing the same superscript within the same column are not different (p>0.05).

**Table 9:** Effects of interaction of treatment and gender on tenderness of meat

Treatment	Female	Male
0	2.85^a^	3.38^ab^
50	3.03^a^	3.50^a^
100	3.70^b^	3.55^a^
150	3.65^b^	2.83^b^
Standard error	0.233	0.233

a,b Means sharing the same superscript within the same column are not different (p>0.05).
